# Quantitative and qualitative differences in subcutaneous adipose tissue stores across lipodystrophy types shown by magnetic resonance imaging

**DOI:** 10.1186/1471-2342-7-3

**Published:** 2007-03-12

**Authors:** Salam A Al-Attar, Rebecca L Pollex, John F Robinson, Brooke A Miskie, Rhonda Walcarius, Cynthia Harper Little, Brian K Rutt, Robert A Hegele

**Affiliations:** 1 Vascular Biology Research Group, Robarts Research Institute, London, Ontario, N6A 5K8, Canada; 2 Imaging Research Laboratories, Robarts Research Institute, London, Ontario, N6A 5K8, Canada; 3 Department of Medicine, Schulich School of Medicine and Dentistry, University of Western Ontario, London, Ontario, N6A 5A5, Canada; 4 Blackburn Cardiovascular Genetics Laboratory, Robarts Research Institute, 100 Perth Drive, Room 406, London, Ontario, N6A 5K8, Canada

## Abstract

**Background:**

Lipodystrophies are characterized by redistributed subcutaneous fat stores. We previously quantified subcutaneous fat by magnetic resonance imaging (MRI) in the legs of two patients with familial partial lipodystrophy subtypes 2 and 3 (FPLD2 and FPLD3, respectively). We now extend the MRI analysis across the whole body of patients with different forms of lipodystrophy.

**Methods:**

We studied five subcutaneous fat stores (supraclavicular, abdominal, gluteal, thigh and calf) and the abdominal visceral fat stores in 10, 2, 1, 1 and 2 female subjects with, respectively, FPLD2, FPLD3, HIV-related partial lipodystrophy (HIVPL), acquired partial lipodystrophy (APL), congenital generalized lipodystrophy (CGL) and in six normal control subjects.

**Results:**

Compared with normal controls, FPLD2 subjects had significantly increased supraclavicular fat, with decreased abdominal, gluteal, thigh and calf subcutaneous fat. FPLD3 subjects had increased supraclavicular and abdominal subcutaneous fat, with less severe reductions in gluteal, thigh and calf fat compared to FPLD2 subjects. The repartitioning of fat in the HIVPL subject closely resembled that of FPLD3 subjects. APL and CGL subjects had reduced upper body, gluteal and thigh subcutaneous fat; the APL subject had increased, while CGL subjects had decreased subcutaneous calf fat. Visceral fat was markedly increased in FPLD2 and APL subjects.

**Conclusion:**

Semi-automated MRI-based adipose tissue quantification indicates differences between various lipodystrophy types in these studied clinical cases and is a potentially useful tool for extended quantitative phenomic analysis of genetic metabolic disorders. Further studies with a larger sample size are essential for confirming these preliminary findings.

## Background

Adipose tissue distribution is an important biomarker associated with metabolic risk [1]. It is important to develop robust high-resolution quantitative non-invasive imaging technologies for both clinical evaluation and research applications of obesity and related metabolic disorders. Magnetic resonance imaging (MRI) technology holds a great promise as a sensitive, replicable, non-invasive and non-irradiating method to image adipose tissue quantity and distribution [1-3]. As with any quantitative methodology, performance must be evaluated at extreme levels of the study analytes. In this regard, lipodystrophy syndromes represent extreme "experiments of nature" [4-7] that can help test the limits of the resolution of MRI for detecting and quantifying subcutaneous fat.

Lipodystrophies are a heterogeneous group of adipose tissue disorders that can be either inherited or acquired [4-8]. They are characterized by selective loss of fat from different anatomical regions [4,8]. Inherited lipodystrophy syndromes include partial lipodystrophy subtypes 2 and 3 (FPLD2 and FPLD3), which are caused by heterozygous mutations in *LMNA *and *PPARG *genes, respectively, and congenital generalized lipodystrophy (CGL) subtypes 1 and 2, which are caused by homozygous mutations in *AGPAT2 *and *BSCL2 *genes, respectively [4,9]. Acquired forms of lipodystrophy include acquired partial lipodystrophy (APL), which is sometimes associated with heterozygous mutations in the *LMNB2 *gene [10]. Detailed MRI assessments of adipose tissue distribution in these diverse lipodystrophy types have not yet been reported.

We previously reported pilot studies of adipose tissue quantification in the lower extremity from MR images of one patient with FPLD2 and one patient with FPLD3 [11]. While adipose tissue stores in the thigh and calf were markedly reduced in both patients, attenuation of adipose tissue appeared greater in the FPLD2 patient. That small pilot study provided proof-of-principle that combining MRI with semi-automated image analysis could reveal differences even at the extreme low end of the range of fat distribution [11]. We now apply this method to define quantitative and qualitative differences in fat distribution across a range of clinical lipodystrophy syndromes and healthy female controls.

## Methods

### Clinical assessment

We studied a total of 22 female subjects: 16 were patients with various forms of lipodystrophy and 6 were normal healthy controls, with normal fat distribution clinically and absence of mutations in known lipodystrophy genes. The lipodystrophy patients included 10 subjects with FPLD2, two with FPLD3, one with human immunodeficiency virus (HIV)-associated partial lipodystrophy (HIVPL), one with acquired partial lipodystrophy (APL) and two with congenital generalized lipodystrophy (CGL). All patients consented to provide their medical history, underwent physical examination and provided fasting blood samples. All subjects gave informed consent and the University of Western Ontario Institutional Review Board approved the protocol (#11244).

### Bioimpedance analysis

Bioimpedance analysis (BIA) measurements were determined using the BC-418 Segmental Body Composition Analyzer (Tanita, Arlington Heights, IL) which provided percent fat estimates for the total body and lower extremities. The intra-observer coefficient of variation for BIA measurements was 4.5%.

### Molecular screening

DNA was prepared from peripheral blood leukocytes (Puregene, Gentra Systems, Minneapolis, MN). DNA sequences of all subjects were screened for mutations in candidate lipodystrophy genes, namely *LMNA*, *PPARG*, *LMNB2*, *AGPAT2 *and *BSCL2*, which are causative for FPLD2, FPLD3, APL and the two forms of CGL, respectively (MIM 150330, 601487, 150341, 603100, 606158). Amplified and purified genomic DNA fragments were sequenced using reported exon-specific primers for *LMNA *[12], *PPARG *[5,13], *LMNB2 *[10], *BSCL2 *and *AGPAT2 *[14]. Electropherograms were then analyzed using Sequence Navigator v.1.0.1 (Applied Biosystems, Mississauga, ON).

### Magnetic resonance imaging

We used scanning and raw MR image acquisition parameters as reported previously [11]. MRI scans were performed on a 1.5T GE MR Medical system (Model: Signa Excite). A neurovascular array coil was used to image the supraclavicular region and an 8-channel receive-only torso array coil was used to image other body regions.

### Validation of adipose tissue quantification method

**Figure 1:**
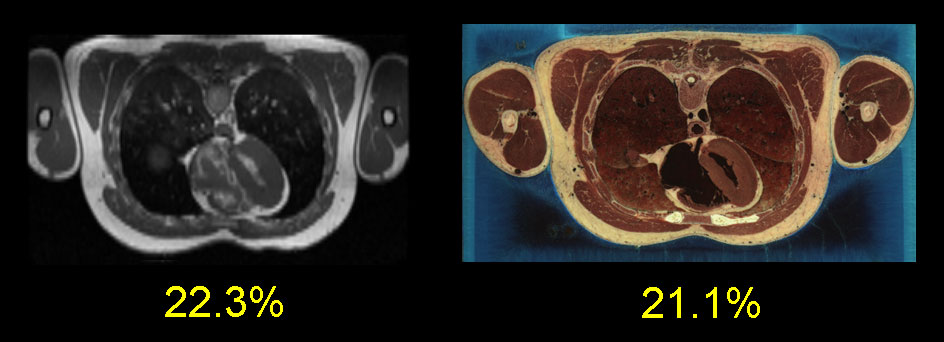
**Validation of adipose tissue quantification method against an anatomical reference standard from the Visible Human Project**. The image on the left is an abdominal transaxial MR image allocated at the upper liver lobe of the Visible Human Male [15, 16]. The image on the right is the corresponding cryosection image obtained from the same subject. Quantified percent subcutaneous adipose tissue values for both images are reported below the respective image.

In order to validate the developed adipose tissue quantification method [11] against a reference anatomical standard, MR and cryosection abdominal images of the male cadaver from the Visible Human Project were analyzed (Figure 1) [15]. Both image types were obtained from the Visible Human online database [16]. Cryosection images were available as 1 mm thick transaxial slices spanning the entire body. MR images of the visible human were available as sagittal slice images. Five transaxial images were selected from the abdominal cryosection images for adipose tissue analysis. These images were anatomically allocated using the following reference points: 1) upper lobe of the liver, 2) upper lobe of the left kidney, 3) upper lobe of the right kidney, 4) lower lobe of the left kidney, 5) lower lobe of the right kidney. In order to obtain the corresponding MR transaxial images, the abdominal MR sagittal image stack was imported into the ImageJ 1.34 n program [17] and re-sliced into 1 mm thick transaxial slice images using the Stack-Reslice tool. Corresponding MR images were selected based on the same anatomical allocations discussed above.

After obtaining the images above, subcutaneous adipose tissue was quantified in both MR and corresponding cryosection images using the method developed in our laboratory [11]. The Pearson correlation coefficient was used to test both intra-observer variation among duplicate blinded MRI and cryosection subcutaneous adipose tissue measurements and also to test the accuracy of fat quantification in MR images in comparison to the anatomically matched cryosection images.

### Adipose tissue quantification

**Figure 2:**
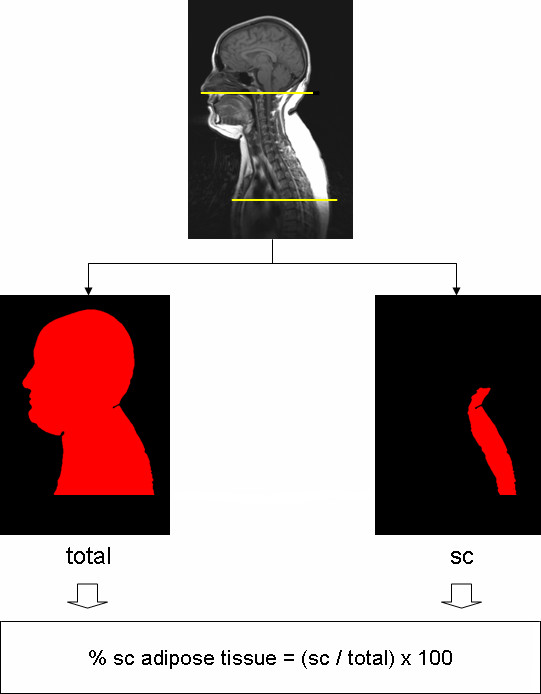
**Quantification of percent adipose tissue in upper back/shoulder region**. For a given sagittal mid-slice MRI image, both the total volume and the subcutaneous (sc) fat volumes were selected using the Connected Threshold Grower tool. Subcutaneous adipose tissue (sc) to be quantified was pre-determined by truncating the raw MR image with upper and lower black-colored lines which extend horizontally from the tip of the nose to the back of the neck and horizontally from the lowermost point of the twelfth spinal vertebra (illustrated by the yellow lines in the top image). The corresponding volumes were determined using the Voxel Counter tool. The percent subcutaneous adipose tissue was calculated for by dividing the voxel count determined for the sc fat by the total voxels for the slice.

**Figure 3:**
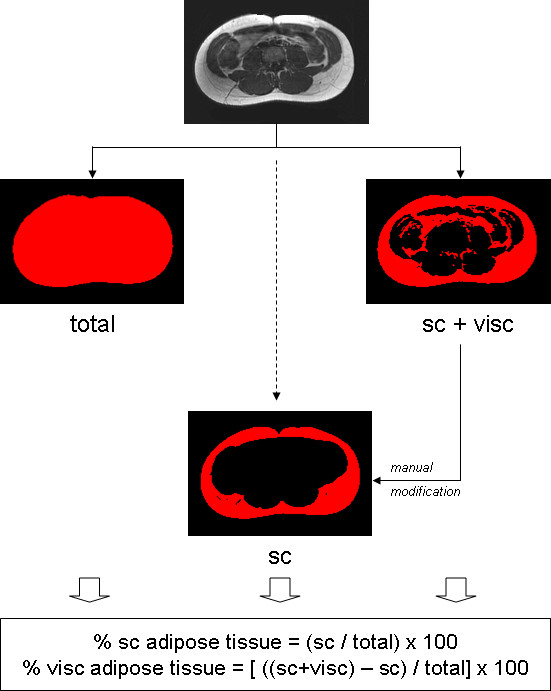
**Quantification of percent adipose tissue in abdomen-L4 region**. For a given transaxial MR image, both the total volume and the total subcutaneous and connected visceral (sc + visc) fat volumes were selected using the Connected Threshold Grower tool. Subcutaneous adipose tissue (sc) cannot be obtained directly due to software limitation, and is selected for by manually eliminating visceral signals present in (sc + visc) images Their corresponding volumes were determined using the Voxel Counter tool. The percent subcutaneous adipose tissue was calculated for by dividing the voxel count determined for the sc fat by the total voxels for the slice. The percent visceral adipose tissue was calculated for by subtracting the sc fat voxel count from that of sc + visc and then dividing the value obtained by the total voxels for the slice. Solid lines in the figure represent direct automated attainment of threshold image via ImageJ program. Dotted lined represent indirect attainment of threshold image, requiring manual modification.

Subcutaneous adipose tissue was quantified from MR images of: 1) mid-line sagittal sections of the supraclavicular region; and transverse sections of the: 2) abdomen at L4 level; 3) gluteal region at the level of the femoral heads; 4) mid-thigh level; and 5) mid-calf level. Supraclavicular fat pad quantification required standardized manual truncation of the raw MR fat signal superiorly and inferiorly along horizontal planes extended parallel to the plane of the scan from the tip of the subject's nose and to the bottom of the twelfth spinal vertebra, respectively (Figure 2). Quantification of subcutaneous adipose and visceral fat at the abdomen-L4 level and gluteal region also required manual truncation: fat adjacent to the viscera within the peritoneal compartment was outlined (Figure 3). Quantification of visceral adipose tissue in abdomen-L4 and gluteal segments was then calculated by subtracting the subcutaneous adipose tissue signal from total subcutaneous and visceral signals and dividing by the total threshold signal (Figure 3). Semi-automated quantification was performed using image analysis software employing Connected Threshold Grower and Voxel Counter tools [11].

### Statistical analysis

All statistical analyses were performed using SAS version 9.2 (SAS Institute, Cary, NC). Mean values between FPLD2 and control groups were compared using student t-tests. A nominal *P*-value < 0.05 was chosen as the threshold for significance for all statistical comparisons. Pearson correlation coefficients were calculated between percent fat in the lower limb as determined by BIA and MRI methods. The Pearson correlation coefficient was also used to test variation among duplicate blinded subcutaneous adipose tissue measurements made by the same observer on different days in the supraclavicular, abdominal-subcutaneous, abdominal-visceral and gluteal MRI sections (intra-observer variability) and also to test inter-observer variation for the same samples, analyzed by a different observer on different days.

## Results

### Clinical, biochemical and anthropometric data

Table 1 shows demographic baseline clinical and biochemical data for study subjects. The mean values for the normal control subjects would not meet any threshold for the National Cholesterol Education Program (NCEP) criteria for the metabolic syndrome. In contrast, mean values, or single values for those disease categories with only a single patient, indicate that all lipodystrophy patient groups would meet the NCEP criteria for the metabolic syndrome [18].

### Genetic analysis

FPLD2 subjects were each heterozygous for the *LMNA *R482Q missense mutation [12]. FPLD3 subjects were heterozygous for the *PPARG *F388L mutation [13]. No mutations were found in any of the candidate genes in the genome of the HIVPL subject. The APL subject was heterozygous for the *LMNB2 *R215Q missense mutation [10]. The CGL subjects were each homozygous for the frameshift mutation in *BSCL2 *[14].

### Validation of adipose tissue quantification method

Subcutaneous adipose tissue values were obtained from 5 transaxial locations in the Visible Human male [15,16] for both MR and cryosection images (Figure 1). The intra-observer correlation coefficients, determined by comparing duplicate blinded measurements of percent subcutaneous fat from the 5 locations, were 0.996 and 0.999 for the MR and cryosection image sets, respectively. The correlation coefficient was 0.999 for the comparison of mean percent subcutaneous adipose tissue values obtained from the MR images and matching cryosection images.

### Intra- and inter-observer correlations for quantitative MRI analysis

Intra- and inter-observer correlations for analysis of the mid-calf and mid-thigh were determined previously [11]. Analysis of adipose tissue in the newly investigated compartments, namely the supraclavicular, abdominal-subcutaneous, abdominal-visceral and gluteal sections, was conducted by two independent observers. Intra- and inter-observer correlation coefficients were determined by comparing two replicates from eight study subjects. The intra-observer correlation coefficients were, on average, 0.994, 0.999, 0992, and 0.999 for the supraclavicular, abdominal-subcutaneous, abdominal-visceral and gluteal sections, respectively. The inter-observer correlation coefficients were, on average, 0.997, 0.995, 0.992, and 0.998 for the supraclavicular, abdominal-subcutaneous, abdominal-visceral and gluteal sections, respectively.

### Correlation of percent fat in thigh determined by BIA and MRI

**Figure 4:**
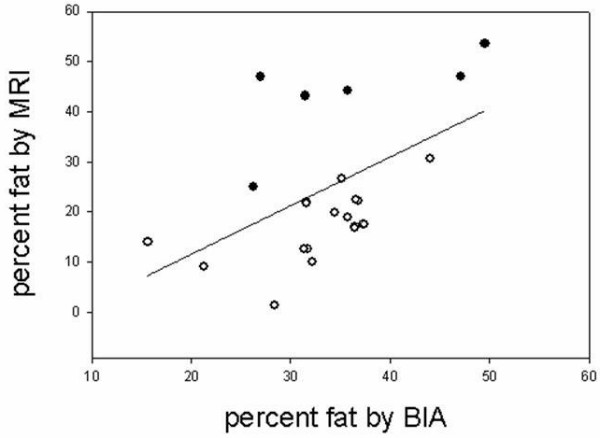
**Correlation of percent fat from mid-thigh obtained by BIA and MRI**. These values were obtained across all subjects in the study. Filled circles represent the control group, while non-filled circles represent the lipodystrophic group. The Pearson correlation coefficient was 0.52 (P < 0.0001).

Figure 4 shows a significant correlation between percent fat as determined in the thigh by MRI and by BIA (P < 0.0001), suggesting that BIA can serve as a rough surrogate for MRI quantification of subcutaneous fat. Although the correlation coefficient of 0.52 was significant, it is still relatively modest, indicating that the variables are somewhat interrelated, but not interchangeable. Interestingly, almost all the data points for lipodystrophy subjects fell below the line of best fit, suggesting that MRI quantification of subcutaneous fat might systematically underestimate total fat content (including intramuscular fat), as estimated by BIA in these subjects.

### MRI quantification of regional fat stores

**Figure 5:**
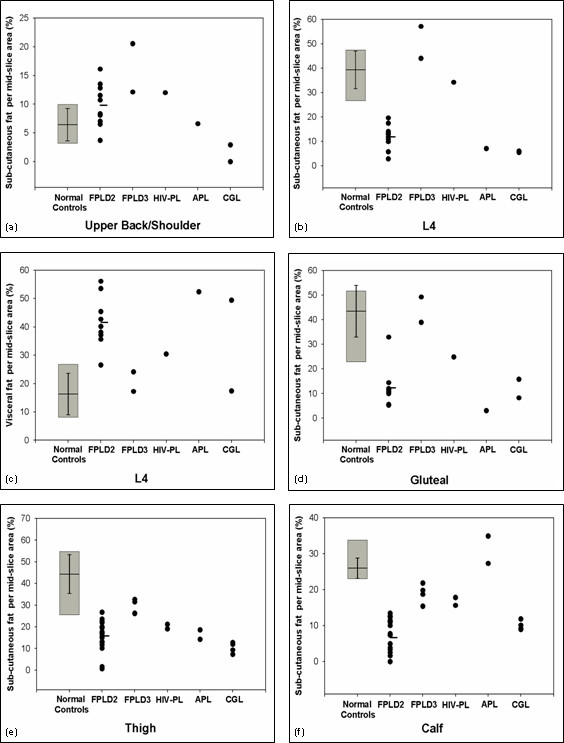
**Percent subcutaneous and visceral adipose tissue values in different body segments of patients and healthy controls**. Shaded region represents the range of adipose tissue values of the normal control group. Horizontal line and whiskers represent the mean and standard deviation of adipose tissue percentages seen in normal controls. Horizontal line in FPLD2 plots represents the mean value of adipose tissue in these subjects. (a) Percent subcutaneous adipose tissue in the upper back and shoulders. (b) Percent subcutaneous adipose tissue in the abdomen-L4 region. (c) Percent visceral adipose tissue in the abdomen-L4 region. (d) Percent subcutaneous adipose tissue in the gluteal region. (e) Percent subcutaneous adipose tissue in the thighs. Values for left and right thigh are plotted as two separate points for each subject. (f) Percent subcutaneous adipose tissue in the calves. Values for left and right calf are plotted as two separate points for each subject.

Figure 5 summarizes the fat quantification experiments. Contrasts of either "increased" or "decreased" fat stores are with reference to the normal ranges for control subjects. Table 2 shows that FPLD2 subjects had significantly increased supraclavicular subcutaneous fat and abdomen-L4 visceral fat, with significantly decreased abdomen-L4, gluteal, thigh and calf subcutaneous fat compared with control subjects (unpaired t-tests, all P < 0.01).

Both FPLD2 and FPLD3 subjects had increased supraclavicular fat (Figure 5a), reflecting the increase in upper body fat observed clinically [19]. The patient with HIVPL also had increased supraclavicular fat, comparable to the FPLD3 patients (Figure 5a). In contrast, supraclavicular fat in the APL subject was not increased and CGL subjects had reduced supraclavicular fat (Figure 5a), consistent with generalized depletion of body fat [4,20].

FPLD2 and FPLD3 subjects had clear disparities in abdomen-L4 subcutaneous fat (Figure 5b), with a significant decrease and no decease in FPLD2 and FPLD3 subjects, respectively. HIVPL, APL and CGL subjects had varying amounts of subcutaneous adipose tissue at the abdomen-L4 level: no decrease for the HIVPL subject, but markedly decreased for the APL and CGL subjects. Again, the HIVPL patient most closely resembled the FPLD3 subject.

Further disparities between FPLD2 and FPLD3 were seen for visceral adipose tissue at the abdomen-L4 level; visceral fat content was the opposite of the subcutaneous fat distribution at this level (Figure 5c), with a significant increase and no increase in the FPLD2 and FPLD3 subjects, respectively. Thus FPLD2 subjects have more severe depletion of abdominal subcutaneous fat and more pronounced accumulation of visceral fat than FPLD3 subjects. The HIVPL patient had marginally increased visceral adipose tissue, again comparable to the FPLD3 subjects. The APL subject had markedly increased visceral adipose tissue while the CGL subjects showed a range of visceral fat.

Gluteal subcutaneous fat distribution was comparable to the abdomen-L4 distribution (Figure 5d). Gluteal fat in the FPLD2 and FPLD3 subjects was decreased and not decreased, respectively (Figure 5d). The HIVPL subject again closely resembled the FPLD3 subjects (Figure 5d). APL and CGL subjects both showed decreased gluteal fat, with APL having the lowest value (Figure 5d).

The most consistently altered fat depot across all lipodystrophy types was decreased subcutaneous thigh fat (Figure 5e). Similar to other subcutaneous depots, FPLD2 subjects had pronounced depletion of subcutaneous thigh fat, while FPLD3 subjects had less depletion of subcutaneous thigh fat (Figure 5e). A descending trend of adipose tissue amount was observed for subjects with HIVPL, APL and CGL, respectively (Figure 5e). Subcutaneous adipose tissue in HIVPL subject most resembled that of FPLD3 subjects. CGL subjects had nearly no subcutaneous adipose tissue in this region and harbored some of the lowest values from these experiments (Figure 5e).

The pattern of subcutaneous fat distribution in the mid-calf was very similar to that observed in the mid-thigh (Figure 5f). FPLD2 subjects had greater depletion of calf fat than FPLD3 subjects (Figure 5f). The HIVPL patient had reduced fat comparable to the quantity seen in FPLD3 (Figure 5f). In contrast to all other lipodystrophic subjects studied, the APL subject had increased adipose tissue in the calves (Figure 5f). CGL subjects showed the greatest decrease in calf fat (Figure 5f).

## Discussion

In this study of all female subjects we have shown that MRI analysis with quantification of fat depots can reveal differences between patients with various forms of lipodystrophy. Evaluating lipodystrophy patients allowed for assessment of the lower limits of resolution of the method. The main findings include: 1) demonstration of significant differences from controls in FPLD2 subjects, with increased suprvaclavicular and visceral fat and decreased subcutaneous abdominal, gluteal, thigh and calf fat; 2) confirmation of the subjective clinical impression of differences between FPLD2 and FPLD3 subjects, such as no increase in visceral fat, no decrease in abdomen-L4 subcutaneous fat and less dramatic depletion of subcutaneous gluteal, thigh and calf fat in FPLD3 compared with FPLD2 subjects; 3) fat repartitioning in the HIVPL subject resembled the FPLD3 subjects; 4) reduced subcutaneous fat in the upper body, gluteal region and thigh, in both APL and CGL subjects, with increased and decreased calf fat in APL and CGL subjects, respectively; and 5) consistently reduced fat in the mid-thigh across the various lipodystrophy types, suggesting that this analyte could help distinguishing whether a form of lipodystrophy might be present in a patient with in whom the clinical diagnosis is unclear.

Sampling measurements of adipose tissue in specific body regions was an important approach for the identification of adipose tissue distribution differences among lipodystrophic patients. Qualitatively, MR images as well as external phenotypes of different lipodystrophic subjects hint to the loss of fat is specific body regions, such as loss of fat in the extremities and general abdominal-visceral fat accumulation that is seen in the FPLD subjects. Whole-body measurements of adipose tissue [21,22], is a potential next step in similar future research. While valuable in its ability to represent total body fat, we chose to implement a sampling approach instead of whole-body fat quantification in this pilot study to help focus on the quantitative investigation of adipose tissue distribution.

We observed very high intra- and inter-observer correlation values for the combination of methods, and furthermore, validated the method against an anatomical reference standard. In addition to reproducibility, the described methods yield results quickly and accurately, with minimal user intervention. While the correlation coefficient of percent fat values found by MRI and BIA was statistically significant, the r of 0.52 indicated only modest correlation, suggesting that the two determinations are not interchangeable. BIA appears to measure total limb fat, including both subcutaneous and intramuscular fat, while MRI measures only subcutaneous fat. Acquisition of such values from a larger number of patients with various lipodystrophy subtypes would verify the results observed here. Future application of this quantification method, or another [23-27], may include quantification of fat depots for "garden variety" obesity, metabolic syndrome or diabetes. This approach might also be applicable to quantify metabolically important substrata of fat.

Widespread application of MRI-based fat quantification would require the development of standards with respect to regions surveyed, anatomical landmarks, number of measurements, etc – similar to the consensus standards agreed upon for carotid intima-media thickness measurements using ultrasound. Regional distribution could be an additional MRI analyte that could be considered together with other intermediate traits in subjects with FPLD or even common metabolic syndrome. Quantification of fat depots using MRI and appropriate image analysis software could provide complementary analytes for research and perhaps eventually for the diagnosis and monitoring of interventions.

## Conclusion

In summary, we report the expanded use of MRI and image analysis software employing Connected Threshold Grower and Voxel Counter tools to quantify fat depots at multiple sites in patients with several different molecular forms of lipodystrophy. The measurements confirmed the clinical impression that FPLD2 and FPLD3 differ with respect to the extent of subcutaneous fat loss; specifically, subcutaneous fat loss below the umbilicus and visceral fat accumulation in FPLD2 are both greater than in FPLD3. Increasing the sample size of subjects with other forms of lipodystrophy will allow for statistical comparisons. These tools can be applied and might prove useful in quantitative phenotype analysis of other forms of lipodystrophy and in less extreme disorders of fat redistribution or repartitioning, such as "garden variety" or common obesity, insulin resistance, or type 2 diabetes.

## Competing interests

The author(s) declare that they have no competing interests.

## Authors' contributions

SAA participated in the experimental design, data acquisition and analysis, interpretation of results, and manuscript writing. RLP participated in the analysis of the MRI data and manuscript writing. JFR participated in data acquisition, analysis and interpretation of results. BAM was involved in the clinical assessment. CHL and RW performed the MRI scans. BKR participated in the experimental design, data analysis, and interpretation of results. RAH participated in the experimental design, data analysis and interpretation of results and manuscript writing. All authors approved the final manuscript.

## Pre-publication history

The pre-publication history for this paper can be accessed here:



## Figures and Tables

**Table 1:** Clinical and biochemical profile of 22 female subjects

	**normal controls**	**FPLD2**	**FPLD3**	**HIVPL**	**APL**	**CGL**
number	6	10	2	1	1	2
age (years)	40 ± 16	48 ± 16	36 ± 15	41	64	40 ± 1
BMI (kg/m^2^)	26.4 ± 6.73	24.3 ± 2.07	33.9 ± 0.72	28.3	20.3	23.0 ± 0.08
Molecular genetic basis of disease	None	*LMNA *R482Q (all)	*PPARG *F388L (all)	None	*LMNB2 *R215G	*BSCL2 *frameshift (both)
diabetes	0/6	3/10	1/2	0/1	1/1	2/2
hypertension	1/6	5/10	2/2	1/1	1/1	1/1
dyslipidemia	0/6	10/10	2/2	1/1	1/1	1/1
total cholesterol (mM)	4.41 ± 0.54	4.76 ± 1.13	3.80 ± 0.43	5.37	3.63	4.08 ± 0.78
triglycerides (mM)	1.08 ± 0.63	2.62 ± 1.09	1.84 ± 1.27	6.29	2.82	2.13 ± 0.83
LDL cholesterol (mM)	2.36 ± 0.33	2.59 ± 1.11	2.42 ± 0.59	ND	1.50	2.09 ± 0.69
HDL cholesterol (mM)	1.56 ± 0.45	1.01 ± 0.29	0.88	0.50	0.85	1.04 ± 0.44
fasting blood glucose (mM)	4.71 ± 1.14	6.01 ± 1.74	8.35 ± 0.21	5.30	12.6	6.65 ± 1.34
insulin (uIU/mL)	13.8 ± 13.7	19.0 ± 14.6	29.5 ± 14.9	27.8	63.6	140 ± 130
apolipoprotein B (g/L)	0.71 ± 0.12	0.90 ± 0.18	ND	ND	0.75	0.99 ± 0.43
apolipoprotein A-I (g/L)	1.51 ± 0.38	1.25 ± 0.28	ND	ND	1.06	1.04 ± 0.21
systolic blood pressure (mm/Hg)	119 ± 19.2	127 ± 13.7	134 ± 8.49	126	108	113 ± 7
diastolic blood pressure (mm/Hg)	70 ± 11.7	73.5 ± 8.9	71.0 ± 8.5	82	56.0	75.0 ± 7.1
waist circumference (cm)	87.0 ± 19.2	87.5 ± 7.9	118 ± 17	107	118	77.5 ± 0.7

Abbreviations: FPLD2 and FPLD3, Dunnigan variety familial partial lipodystrophy types 2 and 3, respectively; HIVPL, Human immunodeficiency virus partial lipodystrophy; APL, acquired partial lipodystrophy; CGL, congenital generalized lipodystrophy

**Table 2:** Comparison of regional mean ± standard deviation percent fat in 10 FPLD2 subjects and 6 normal control subjects

Fat Depot	FPLD2	Control	P-value
Subcutaneous			
Supraclavicular	7.34 ± 3.40	4.10 ± 2.42	0.049
Abdomen – L4	8.27 ± 4.98	30.9 ± 8.00	< 0.0001
Gluteal	12.4 ± 7.70	29.8 ± 10.4	0.0004
Thigh	12.7 ± 6.70	38.0 ± 9.80	< 0.0001
Calf	4.34 ± 4.94	25.4 ± 3.50	< 0.0001
Visceral			
Abdomen – L4	35.4 ± 8.60	8.81 ± 7.15	< 0.0001
